# Mesogenic Polyelectrolyte Gels Absorb Organic Solvents and Liquid Crystalline Molecules

**DOI:** 10.3390/polym8040148

**Published:** 2016-04-19

**Authors:** Yusuke Nishikori, Kazuya Iseda, Kenta Kokado, Kazuki Sada

**Affiliations:** 1 Graduate School of Chemical Sciences and Engineering, Hokkaido University, Kita10 Nishi8, Kita-ku, Sapporo 060-0810, Japan; katsuobushi@frontier.hokudai.ac.jp (Y.N.); i-kzy@mail.sci.hokudai.ac.jp (K.I.); 2 Department of Chemistry, Faculty of Science, Hokkaido University, Kita10 Nishi8, Kita-ku, Sapporo 060-0810, Japan

**Keywords:** liquid crystal, polymer gel, polyelectrolyte

## Abstract

In this paper, mesogenic polyelectrolyte gels (MPEgels) tethering mesogenic groups on the side chains were synthesized from a mesogenic monomer and ionic monomer via a conventional radical polymerization process. The obtained MPEgels absorbed various organic solvents in a wide range of dielectric constants from chloroform (ε = 7.6) to DMSO (ε = 46.5). The electrostatic repulsion among the polymer chains and the osmotic pressure between the interior and exterior of the MPEgel is responsible for the high swelling ability, revealed by the common ion effect using tetra(*n*-hexyl)ammonium tetra(3,5-bis(trifluoromethyl)phenylborate (**THATFPB**). The obtained MPEgels could also absorb liquid crystalline molecules such as 4-cyano-4’-pentylbiphenyl (**5CB**), analogously caused by the above-mentioned polyelectrolyte characteristic. The MPEgels exhibited liquid crystal transition temperature (*T*_NI_) on differential scanning calorimetry (DSC) measurement, and the increase of the ionic group content lowered *T*_NI_. The MPEgels absorbing liquid crystalline molecules exhibited differing *T*_NI_, dependent on the compatibility of the mesogenic group on the side chain to the liquid crystalline molecule.

## 1. Introduction

Polyelectrolytes are defined as polymers possessing dissociable ionic groups on their main or side chains; thus, they release small ions into the solution, leaving a charged polymer chain [1,2,3,4,5]. In the dissociated state, the polymer chain exhibits an extended form driven by electrostatic repulsion among the charges on the chains. This feature provides various fascinating properties to polyelectrolytes, such as large viscosity, ion exchangeability, ion conductivity, or osmotic pressure, and accordingly they have found many applications including fuel cells [6,7,8,9,10], super absorbent polymers [11,12,13,14,15,16,17], or layer-by-layer membranes [18,19,20,21,22,23,24]. The requirements of chemical structures for a polyelectrolyte are regarded as both (1) dissociable ionic groups and (2) highly compatible polymer chains in a given medium [25], while most research on polyelectrolytes has employed highly polar media such as water, DMSO, or DMF.

By utilizing the features of polyelectrolytes in low polar or nonpolar media, we have recently achieved a new class of polyelectrolyte [26], and developed their applications including super-absorbent polymers for organic solvents [27,28,29,30,31,32,33,34,35], multilayers via layer-by-layer (LbL) deposition in organic solvents [36], polymer brushes [37], and ionomer blends of polystyrene and poly(butyl methacrylate) [38]. Our molecular design simply relies on the introduction of a small amount of tetraalkylammonium tetrakis(3,5-bis(trifluoromethyl)phenyl) borate (TFPB) as a lipophilic and dissociable ionic group onto polymer chains, bringing about utility as a polyelectrolyte in nonpolar media.

As an unexplored solvent for polyelectrolyte gels, in this research, we focused on liquid crystalline molecules. Liquid crystalline molecules are substances in a state that has both properties of liquid fluidity and crystal anisotropy [39,40]. Their attractive properties have been extensively investigated due to their potential application for displays [41,42,43,44], lasers [45,46,47], semiconductors [48], and so on, derived from their stimuli-responsive orientational ability. In addition, liquid crystal is known as an effective solvent, such as for polymerization [49,50], swelling gels [51,52,53,54], and forming nanofibers [55,56], and it offers a special orientation to the produced materials. Despite the characteristic of liquid crystalline molecules as a solvent, no studies have paid attention to the use of polyelectrolyte gels in a liquid crystalline molecule because of the slight compatibility between conventional polyelectrolytes and liquid crystal molecules. In order to address this circumstance, we designed polyelectrolyte gels efficiently functioning in a liquid crystalline molecule by utilizing the lipophilic ion pair described above. In this paper, we prepared mesogenic polyelectrolyte gels tethering mesogenic groups and ionic groups via a conventional radical polymerization process, and investigated their swelling and orientating properties.

## 2. Experimental Section

### 2.1. Materials and Measurements

The ionic monomer, 3-acryloyloxypropyl-tri(n-hexyl)ammonium (**3**), was prepared via reported method [27]. Other reagents and solvents were purchased from commercial sources and used without further purification. For example, 4-cyano-4’-hydroxybiphenyl (>98.0%), 4-cyano-4’-pentylbiphenyl (>98.0%), 5-bromo-1-pentanol (>90.0%), 6-bromo-1-hexanol (>95.0%), acryloyl chloride (>98.0%) and benzoyl peroxide (78.0%) were purchased from Tokyo Chemical Industry Co., Ltd., (Tokyo, Japan) and potassium carbonate (99.5%), *N*,*N*-dimethylformamide (>99.0%), triethylamine (>99.0%), and dichloromethane (>99.0%) was purchased from Wako Pure Chemical Industries, Ltd. (Osaka, Japan) The ^1^H NMR spectra were measured on a JNM-AL300 apparatus (JEOL Ltd., Tokyo, Japan), using 0.05% tetramethylsilane (TMS) as an internal standard. Differential scanning calorimetry (DSC) was conducted by DSC1 Star System (Mettler Toledo International Inc, Greifensee, Switzerland) with heating rate of 5 °C /min under nitrogen atmosphere.

### 2.2. Synthesis of Compound **1**

In a 200 mL round-bottom flask, 4-cyano-4’-hydroxybiphenyl (1.17 g, 6.0 mmol), potassium carbonate (1.65 g, 11.2 mmol), and dry DMF (60 mL) were mixed, and stirred for an hour at 80 °C. Then 5-bromopentanol (1.04 g, 6.2 mmol) was added to the mixture, and stirred additional 10 h at 80 °C. The mixture was cooled to room temperature, and extracted with EtOAc, followed by wash with distilled water. The organic layer was dried with MgSO_4_, and the solvent was removed under vacuum. The residue was recrystallized from toluene to obtain compound **1** as a white solid (1.14 g, 66%). ^1^H NMR (300 MHz, CDCl_3_): δ (ppm) 7.61 (6H, m, Ar–*H*), 6.99 (2H, d, J = 6.7 Hz, Ar–*H*), 4.03 (2H, t, J = 6.5 Hz, ArO–C*H*_2_–), 3.71 (2H, d, J = 4.7 Hz, –C*H*_2_–OH), 1.79 (6H, br, – (C*H*_2_)_3_–), 1.37 (1H, s, –O*H*).

### 2.3. Synthesis of Monomer **M5**

To a solution of compound **1** in dry CH_2_Cl_2_ (200 mL) was added triethylamine (3.95 g, 3.9 mmol) under N_2_ atmosphere. Then acryloyl chloride (3.76 g, 41.5 mmol) was added dropwise, and stirred for 14 h at room temperature. The mixture was washed with NaHCO_3_ aq. and distilled water, and dried with MgSO_4_.

The solvent was removed under vacuum, and the residue was purified by silica gel chromatography (CH_2_Cl_2_) to obtain monomer **M5** as a white solid (2.98 g, 61%). ^1^H NMR (300 MHz, CDCl_3_): δ (ppm) 7.69 (2H, d, J = 8.5 Hz, Ar–*H*), 7.63 (2H, d, J = 8.5 Hz, Ar–*H*), 7.53 (2H, d, J = 8.8 Hz, Ar–*H*), 6.98 (2H, d, J = 8.8 Hz, Ar–*H*), 6.41 (1H, dd, J = 17.2, 1.4 Hz, vinyl–*H*), 6.13 (1H, dd, J = 17.2, 10.4 Hz, vinyl–*H*), 5.83 (1H, dd, J = 10.4, 1.4 Hz, vinyl–*H*), 4.20 (2H, t, J = 6.3 Hz, –COO–C*H*_2_–), 4.02 (2H, t, J = 6.3 Hz, ArO–C*H*_2_–), 1.80 (4H, m, –COOCH_2_–C*H*_2_–, ArOCH_2_–C*H*_2_–), 1.58 (2H, m, –C*H*_2_–).

### 2.4. Synthesis of Compound **2**

The procedure was similar with that for compound **1**, using 6-bromohexanol instead of 5-bromopentanol. ^1^H NMR (300 MHz, CDCl_3_): δ (ppm) 7.69 (2H, d, J = 2.2 Hz Ar–*H*), 7.65 (2H, d, J = 2.2 Hz Ar–*H*), 7.52 (2H, d, J = 8.8 Hz Ar–*H*), 6.99 (2H, d, J = 8.8 Hz Ar–*H*), 4.03 (2H, t, J = 6.5 Hz, ArO–C*H*_2_–), 3.67 (2H, m, –C*H*_2_–OH), 1.83 (2H, quin, J = 7.1 Hz, –CH_2_–C*H*_2_OH), 1.67–1.45 (6H, m, –(C*H*_2_)_3_–), 1.37 (1H, s, –OH).

### 2.5. Synthesis of Monomer **M6**

The procedure was similar with that for compound **M5**. ^1^H NMR (300 MHz, CDCl_3_): δ (ppm) 7.71 (2H, d, J = 8.5 Hz, Ar–*H*), 7.64 (2H, d, J = 8.5 Hz, Ar–*H*), 7.54 (2H, d, J = 8.8 Hz, Ar–*H*), 6.99 (2H, d, J = 8.8 Hz, Ar–*H*), 6.41 (1H, dd, J = 17.4, 1.4 Hz, vinyl–*H*), 6.15 (1H, dd, J = 17.4, 10.4 Hz, vinyl–*H*), 5.82 (1H, dd, J = 10.4, 1.4 Hz, vinyl–*H*), 4.18 (2H, t, J = 6.6 Hz, –COO–C*H*_2_–), 4.01 (2H, t, J = 7.1 Hz, ArO–C*H*_2_–), 1.83 (2H, quin, J = 7.1 Hz, –COOC*H*_2_–CH_2_–), 1.72 (2H, quin, J = 7.1 Hz, ArOCH_2_–C*H*_2_–), 1.50 (4H, m, –(C*H*_2_)_2_–).

### 2.6. Preparation of **MPEG5-p** and **MPEG6-p**

In a test tube, ionic monomer **3** and mesogenic monomer (**M5** or **M6**) were dissolved in DMF (1 M, 120 μL). Then DMF solution of 1,6-hexanediol diacrylate (**HDA**, 0.1 M, 60 μL) and benzoyl peroxide (0.1 M, 60 μL) were added, and the mixture was completely degassed by the freeze–thaw method and sealed. The tube was heated at 80 °C for 24 h. After cooling to room temperature, the isolated gel was thoroughly washed by repeatedly immersing THF, and dried under vacuum.

### 2.7. Preparation of **MPEP5-5**

A linear polymer **MPEP5-5** was synthesized for ^1^H NMR measurement in an essentially same procedure as that for **MPEG5-5**. In a test tube, ionic monomer **3** and mesogenic monomer (**M5**) were dissolved in DMF (1 M, 180 μL). Then DMF solution of benzoyl peroxide (0.1 M, 60 μL) was added, and the mixture was completely degassed by the freeze–thaw method and sealed. The tube was heated at 80 °C for 24 h. After cooling to room temperature, the polymer was purified by repeated reprecipitation in methanol and dried under vacuum. ^1^H NMR (300 MHz, DMF-*d*_7_): δ (ppm) 7.93–7.44 (6H, m, Ar–*H*), 7.12–6.75 (2H, br, Ar–*H*), 4.32–3.70 (4H, m, –C*H*_2_–O–), 2.58–2.22 (1H, br, main chain –C*H*–), 2.05–1.00 (8H, m, –C*H*_2_– (main and side chains)), 0.92–0.70 (0.41H, br, –CH_3_, ionic moiety). The peaks from ionic moiety were difficult to observe besides that of the end methyl group. From the peak, the content of ionic monomer content was determined as 4.4%.

### 2.8. Swelling Degree Measurement

The shrunk gel was immersed in an organic solvent including hexane (ε = 1.9), chloroform (ε = 4.8), THF (ε = 7.6), dichloromethane (ε = 8.9), 1-hexanol (ε = 13.3), cyclopentanone (ε = 13.6), 1-butanol (ε = 17.5), 2-butanone (ε = 18.5), acetone (ε = 20.6), methanol (ε = 32.7), DMF (ε = 36.7), and DMSO (ε = 1.9) for 48 h, then weighed. The swelling degree (*Q*) was determined as follows

$$Q=\;(W_{\text{wet}}-W_{\text{shrunk}})/W_{\text{shrunk}}$$

where *W*_wet_ and *W*_shrunk_ indicate the weight of swollen and shrunk gels, respectively.

## 3. Results and Discussion

**Scheme 1:**
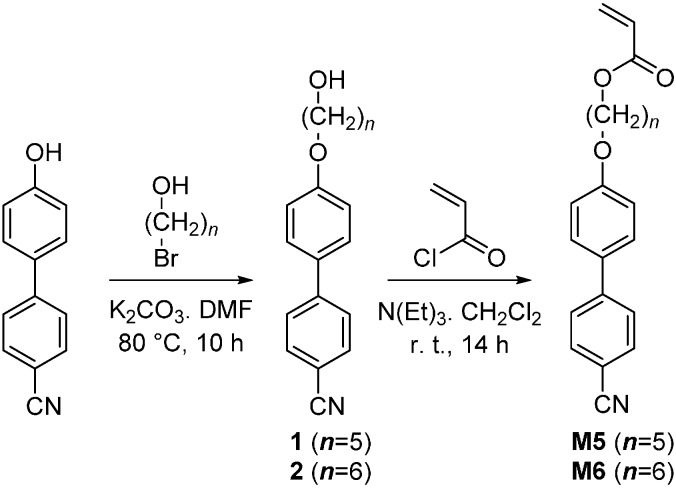
Synthetic route for mesogenic monomers (**M5** and **M6**).

**Scheme 2:**
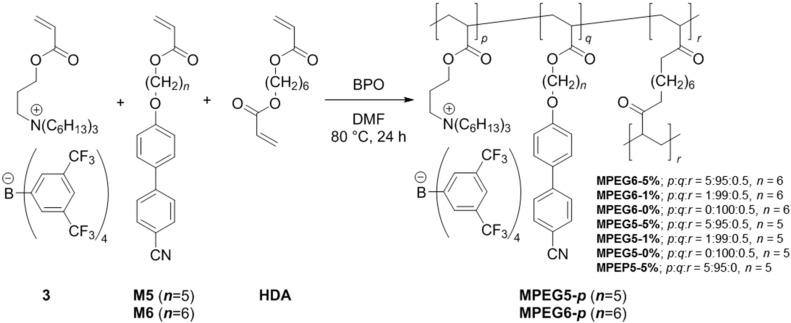
Preparation of the MPEgels (**MPEG5-*p*** and **MPEG6-*p***). HDA: 1,6-hexanediol diacrylate.

**Figure 1:**
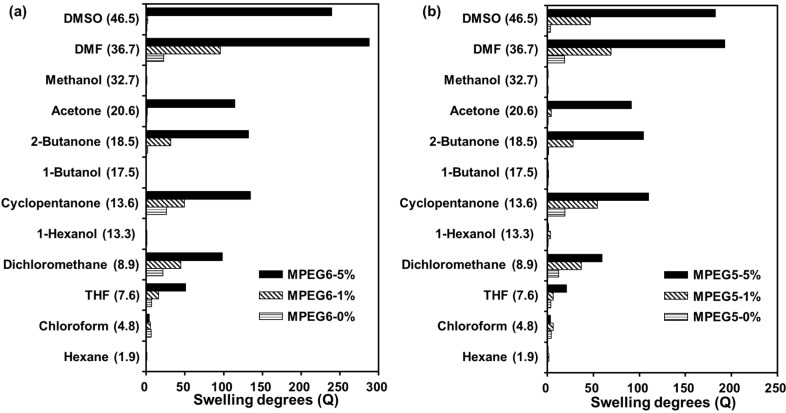
Swelling degrees of (**a**) **MPEG6-*p*** and (**b**) **MPEG5-*p*** in various organic solvents.

The MPEgel tethering mesogenic group on the side chain (**MPEGn-*p***) was prepared via conventional free radical polymerization of the mesogenic monomer (***M*n**, Scheme 1) and ionic monomer (**3**) initiated by benzoyl peroxide (BPO), using 1,6-hexanediol diacrylate (**HDA**) as the crosslinker (Scheme 2). The ionic monomer content is given by *p*, whereas *n* expresses the length of the alkyl linker on the mesogenic monomer. As a result of the ^1^H NMR measurement of a linear polymer (**MPEP5-5**), the ionic monomer content was found to be 4.4%, showing good correlation with the 5% of feed ratio. The swelling degree of obtained MPEgels was investigated in organic solvents with various polarities from hexane (ε = 1.9) to DMSO (ε = 46.5), as shown in Figure 1 and Tables S1 and S2 in the Supplementary Materials. In non-polar solvents such as hexane and chloroform, the MPEgels showed a modest swelling degree, indicating no effect of introduced ionic groups. For example, the swelling degrees of **MPEG6-5%**, **MPEG6-1%**, and **MPEG6-0%** were 0.2, 1.0, and 0.4 in hexane, and 3.7, 5.3, and 6.5 in chloroform (Figure 1a). In these solvents, the extremely low polarity suppressed ionic dissociation of tetraalkylammonium tetrakis(3,5-bis(trifluoromethyl)phenyl) borate (TFPB), forming a tightly bound ion pair or highly aggregated species. Additionally, in alcoholic solvents such as hexanol, butanol, and methanol, the MPEgels were collapsed, due to the low compatibility of the polymer chain to the solvents. On the other hand, in THF, dichloromethane, cyclopentanone, 2-butanone, acetone, DMF, and DMSO, the MPEgels exhibited an obvious increment in swelling degrees driven by the introduction of the ionic group. The swelling degrees of **MPEG6-5%** and **MPEG5-5%** were 51 and 21 in THF, 98 and 59 in dichloromethane, 134 and 110 in cyclopentanone, 132 and 105 in 2-butanone, 114 and 91 in acetone, 288 and 193 in DMF, and 239 and 183 in DMSO. This behavior is attributed to the dissociation of the ion pair to free ions, causing electrostatic repulsion and osmotic pressure for the expansion of the polymer network and absorption of the solvents. These results indicated that the obtained MPEgels can act as a polyelectrolyte gel in various organic solvents by introducing TFPB salts.

In order to confirm the function of the polyelectrolyte, we investigated the common ion effect in the swelling behavior by the addition of tetra(n-hexyl)ammonium tetra(3,5-bis(trifluoromethyl)phenylborate (**THATFPB**) as a low molecular weight electrolyte. In THF, in which MPEgels showed an apparent increment of swelling degree with the introduction of the ionic group, the addition of 1 mM **THATFPB** resulted in decrease of the swelling degree, for example 12 to five in **MPEG6-5%**, six to four in **MPEG6-1%**, four to two in **MPEG5-5%,** and four to three in **MPEG5-1%** (Figure S1). On another front, a smaller effect was observed in DMF, presumably because of the high dissociation degree, even with the existence of the common ion. In chloroform, almost no change was detected due to the slight dissociation degree, due to almost no dissociation even without the common ion. These observations proved that the obtained MPEgels absorbed organic solvents by adopting the same mechanism as that of hydrogels in water.

**Figure 2:**
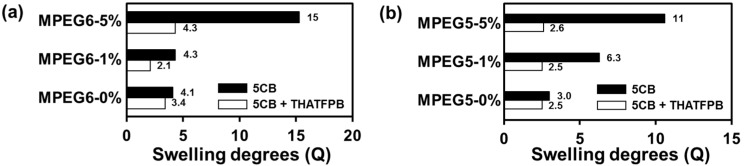
Swelling degree of (**a**) **MPEG6-*p*** and (**b**) **MPEG5-*p*** in a liquid crystalline molecule **5CB** with and without **THATFPB**.

We attested the swelling behavior of the MPEgels in a liquid crystalline molecule by employing 4-cyano-4’-pentylbyphenyl (**5CB**) as a solvent. The **5CB** has an anisotropic molecular geometry, and offers different permittivity dependent on the molecular axis, parallel to the longer axis (εǁ = 17.9) and perpendicular to the longer axis (ε⊥ = 6.9). Compared to nonionic mesogenic gels (**MPEG6-0%**; *Q* = 4.1, **MPEG5-0%**; *Q* = 3.0), MPEgels with high ionic content (**MPEG6-5%**, **MPEG5-5%**) showed higher swelling degrees of 15 and 11, respectively (Figure 2). The MPEgel **MPEG5-1%** showed a moderate increase of swelling degree (*Q* = 6.3), whereas it was not observed in **MPEG6-1%** (*Q* = 4.3), probably due to the higher assembling effect derived from the longer alkyl chain of the mesogenic group with *n* = 6. Furthermore, the addition of **THATFPB** (1 mM) resulted in the collapse of MPEgels, especially with a higher ionic group content. This fact implies the prepared MPEgels can act as a polyelectrolyte gel even in the liquid crystalline molecule.

**Figure 3:**
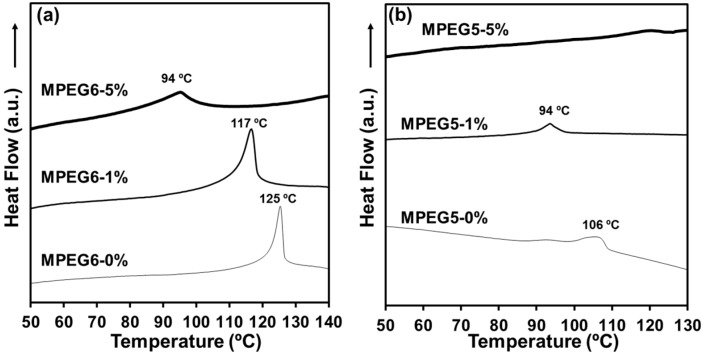
DSC thermograms of the MPEgels (**a**) **MPEG6-*p*** and (**b**) **MPEG5-*p*** upon the cooling process (5 °C/min).

With respect to the liquid crystallinity, differential scanning calorimetry (DSC) was conducted on the obtained MPEgels in a shrunken state (Figure 3). The nonionic mesogenic gels (**MPEG6-0%** and **MPEG5-0%**) exhibited the transition temperature (*T*_NI_) at 125 and 106 °C, respectively. The introduction of ionic groups resulted in a decrease of *T*_NI_, for example 117 °C for **MPEG6-1%** and 94 °C for **MPEG5-1%**. Larger amounts of ionic groups gave rise to a further decrease of *T*_NI_ (94 °C for **MPEG6-5%**) or the disappearance of *T*_NI_ for **MPEG5-5%**. The difference between **MPEG6-5%** and **MPEG5-5%** derived from the higher liquid crystallinity of **MPEG6-5%**, attributed to the odd-even effect of mesogenic molecules [57]. Indeed, 4-cyano-4’-hexyloxybiphenyl (6OCB) shows a higher *T*_NI_ at 76 °C compared to that of 4-cyano-4’-pentyloxybiphenyl (5OCB, *T*_NI_ = 68 °C). The fact that the introduction of ionic groups led to the decrease of *T*_NI_ was also confirmed by the DSC measurement of **5CB** with a certain amount of **THATFPB** (Figure S2), because of the suppression effect of “non-mesogenic” **THATFPB**. The *T*_NI_ of **5CB** originally appears at 34 °C, and the addition of **THATFPB** gradually decreased the *T*_NI_ (23 °C with 50 mM **THATFPB**). Therefore, the introduction of ionic groups was responsible for the *T*_NI_ decrease; however, the liquid crystallinity could be still preserved by proper selection of the structure of the mesogenic or ionic group content. The liquid crystallinity of the MPEgels was also confirmed by polarizing microscopy as shown in Figure S3.

These MPEgels swollen in **5CB** showed different behavior in the DSC measurement (Figure S4). The **MPEG5-*p*** exhibited only one exothermic peak, irrelevant to the original *T*_NI_ of **5CB**. On the other hand, **MPEG6-*p*** exhibited two separated peaks, with low or no ionic group content. These two peaks are assignable to the liquid crystal transition derived from **5CB** (lower *T*_NI_) and the MPEgels (higher *T*_NI_). With the higher ionic group content (**MPEG6-5%**), the two peaks are coalesced, due to the decrease of the *T*_NI_ of the MPEgel. This result also refers to the odd-even effect of the side chain, and thus **MPEG5-*p*** possesses higher interacting ability with **5CB** because of the same length of the alkyl linker.

## 4. Conclusions 

To summarize our results, we successfully prepared mesogenic polyelectrolyte gels tethering mesogenic groups and lipophilic ion groups on the side chains. The obtained MPEgels absorbed various organic solvents in a wide range of dielectric constants from chloroform (ε = 7.6) to DMSO (ε = 46.5), except for extremely nonpolar solvents (ε < 4.8) or alcohols. Their high swelling ability resulted from electrostatic repulsion among the polymer chains and osmotic pressure between the interior and exterior of the MPEgel, revealed by common ion effect using **THATFPB**. Additionally, the MPEgels could absorb liquid crystalline molecules such as **5CB**, also driven by the above-mentioned polyelectrolyte feature. The MPEgels showed liquid crystal transition temperature (*T*_NI_) on the DSC measurement, and an increase of the ionic group content resulted in a decrease of *T*_NI_, due to lowering the liquid crystallinity by the ion-dipole interaction. The MPEgels absorbing liquid crystalline molecules exhibited differing *T*_NI_ dependent on the compatibility of the mesogenic group on the side chain to the liquid crystalline molecule. These findings will allow us to explore liquid crystalline molecules as a medium for polyelectrolytes toward applications including soft actuators, artificial muscles, or nonlinear optical devices.

## Supplementary Materials

Supplementary materials can be found at www.mdpi.com/2073-4360/8/4/148/s1.

## Abbreviations

The following abbreviation is used in this manuscript:

MPE: Mesogenic Polyelectrolyte
