# Supplementary material for: Mesogenic Polyelectrolyte Gels Absorb Organic Solvents and Liquid Crystalline Molecules
Source: Polymers (Basel). 2016 Apr 19;8(4):148. doi: 10.3390/polym8040148 (PMC6432411; doi:10.3390/polym8040148)
Supplement: Supplementary file 1 [file polymers-08-00148-s001.pdf]

Supplementary Materials: Mesogenic Polyelectrolyte Gels Absorb Organic Solvents and Liquid Crystalline Molecules

Yusuke Nishikori, Kazuya Iseda, Kenta Kokado and Kazuki Sada

Table S1. Swelling degrees of the MPEgels (**MPEG6-*p***).

	ε	MPEG6-5%	MPEG6-1%	MPEG6-0%
DMSO	46.5	239	2.0	0.9
DMF	36.7	288	96	23
Methanol	32.7	0.3	0.8	0.5
Acetone	20.6	114	1.5	1.1
2-Butanone	18.5	132	32	1.6
1-Butanol	17.5	0.5	0.6	0.3
Cyclopentanone	13.6	134	49	26
1-Hexanol	13.3	0.5	1.1	0.9
CH_2Cl_2	8.9	98	45	21
THF	7.6	51	16	7.2
$CHCl_3$	4.8	3.7	5.3	6.5
Hexane	1.9	0.2	1.0	0.4

Table S2. Swelling degrees of the MPEgels (**MPEG5-*p***).

	ε	MPEG5-5%	MPEG5-1%	MPEG5-0%
DMSO	46.5	183	46	3.4
DMF	36.7	193	69	19
Methanol	32.7	0.5	0.3	0.5
Acetone	20.6	91	4.0	0.8
2-Butanone	18.5	105	28	1.3
1-Butanol	17.5	0.8	1.2	0.9
Cyclopentanone	13.6	110	54	19
1-Hexanol	13.3	1.2	3.3	0.6
CH_2Cl_2	8.9	59	37	12
THF	7.6	21	6.0	3.5
$CHCl_3$	4.8	3.4	6.3	4.3
Hexane	1.9	0.5	1.7	0.1

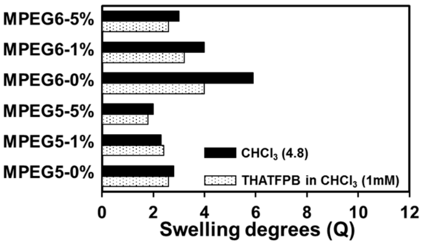

(**a**)

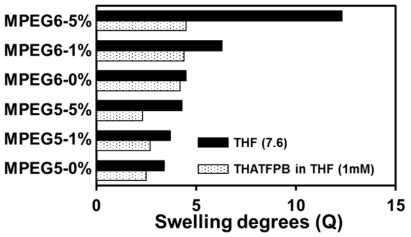

(**b**)

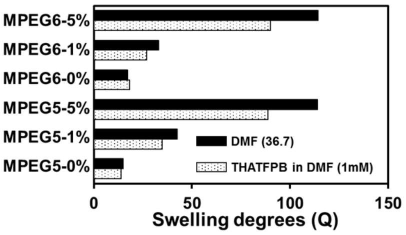

(**c**)

Figure S1. Swelling degrees of the MPEgels with and without the common ion in (**a**) $CHCl_3$; (**b**) THF; and (**c**) DMF.

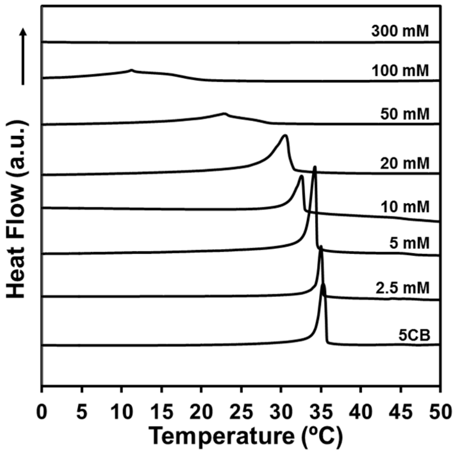

Figure S2. DSC thermogram of **5CB** with certain concentration of **THATFPB** upon the cooling process (5 °C/min).

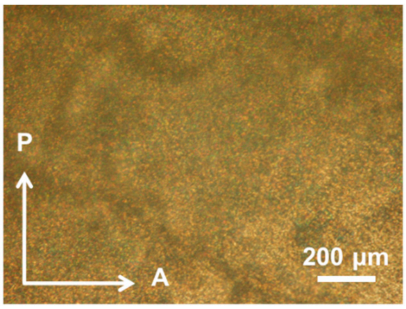

Figure S3. A polarizing micrograph of **MPEG6-5%** in shrunken state at room temperature under crossed-Nicole.

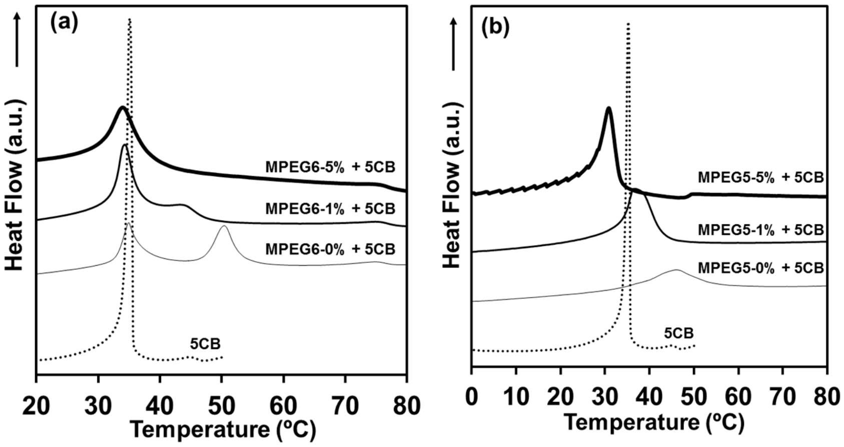

Figure S4. DSC thermograms of MPEgels (**a**) **MPEG6-*p*** and (**b**) **MPEG5-*p*** absorbing **5CB**, upon the cooling process (5 °C/min). A DSC thermogram of **5CB** only is also illustrated for comparison.

© 2016 by the authors; licensee MDPI, Basel, Switzerland. This article is an open access article distributed under the terms and conditions of the Creative Commons Attribution (CC-BY) license (http://creativecommons.org/licenses/by/4.0/).
